# JS-K, a Nitric Oxide Prodrug, Has Enhanced Cytotoxicity in Colon Cancer Cells with Knockdown of Thioredoxin Reductase 1

**DOI:** 10.1371/journal.pone.0008786

**Published:** 2010-01-20

**Authors:** Kornelia Edes, Pamela Cassidy, Paul J. Shami, Philip J. Moos

**Affiliations:** 1 Huntsman Cancer Institute, University of Utah, Salt Lake City, Utah, United States of America; 2 Department of Dermatology, University of Utah, Salt Lake City, Utah, United States of America; 3 Department of Medicinal Oncology, University of Utah, Salt Lake City, Utah, United States of America; 4 Department of Pharmacology and Toxicology, University of Utah, Salt Lake City, Utah, United States of America; 5 Department of Medicinal Chemistry, University of Utah, Salt Lake City, Utah, United States of America; Roswell Park Cancer Institute, United States of America

## Abstract

**Background:**

The selenoenzyme thioredoxin reductase 1 has a complex role relating to cell growth. It is induced as a component of the cellular response to potentially mutagenic oxidants, but also appears to provide growth advantages to transformed cells by inhibiting apoptosis. In addition, selenocysteine-deficient or alkylated forms of thioredoxin reductase 1 have also demonstrated oxidative, pro-apoptotic activity. Therefore, a greater understanding of the role of thioredoxin reductase in redox initiated apoptotic processes is warranted.

**Methodology:**

The role of thioredoxin reductase 1 in RKO cells was evaluated by attenuating endogenous thioredoxin reductase 1 expression with siRNA and then either inducing a selenium-deficient thioredoxin reductase or treatment with distinct redox challenges including, hydrogen peroxide, an oxidized lipid, 4-hydroxy-2-nonenol, and a nitric oxide donating prodrug. Thioredoxin redox status, cellular viability, and effector caspase activity were measured.

**Conclusions/Significance:**

In cells with attenuated endogenous thioredoxin reductase 1, a stably integrated selenocysteine-deficient form of the enzyme was induced but did not alter either the thioredoxin redox status or the cellular growth kinetics. The oxidized lipid and the nitric oxide donor demonstrated enhanced cytotoxicity when thioredoxin reductase 1 was knocked-down; however, the effect was more pronounced with the nitric oxide prodrug. These results are consistent with the hypothesis that attenuation of the thioredoxin-system can promote apoptosis in a nitric oxide-dependent manner.

## Introduction

The mammalian thioredoxin system consists of the selenoprotein thioredoxin reductase (TR), thioredoxin (Trx), and electron donor NADPH. The TR-Trx system participates in diverse redox reactions in cells [1], from supporting DNA synthesis [2] to redox-dependent cell signaling pathways [3]–[6]. Trx and TR may facilitate growth and/or survival of malignant cells as their expression is elevated in some tumors [7], [8]. Thioredoxin reductase enzymatic activity is not limited to thioredoxin, instead, many substrates have been identified, including; selenocompounds, ascorbate, lipoate, and oxidized lipids [9]–[11]. However, some oxidized lipids function to inhibit TR1 activity by reacting with the nucleophilic C-terminus that includes the Sec residue [12]–[14].

The adjacent selenocysteine (Sec) and cysteine (Cys) residues in the C-terminus of mammalian TRs are required for reductase activity when Trx is the substrate; however, Sec-deficient TR1 may have biochemical [15] and biological [16] activities distinct from Sec-sufficient TR1 that may be relevant in cancer or other disease. Sec-deficient TR1 has demonstrated pro-apoptotic activity in studies evaluating the role of TR1 in interferon and retinoic acid-induced apoptosis [17], as well as more recent supporting data that has demonstrated Sec-deficient TR1 species (designated SecTRAPs) are by themselves potent initiators of apoptosis in human cancer cell lines [16]. Apoptosis in these cases were hypothesized to be mediated by increased oxidative stress in the cells. These examples suggest that disruption of the C-terminus of TR1 results in a gain-of-function protein that might be a useful pro-apoptotic agent if it could be targeted to malignant cells.

In this study we have examined the effects on colon cancer cells of two scenarios in which canonical TR1 activity (i.e. the ability to reduce Trx) has been mitigated either by siRNA treatment or mutation of the C-terminal Sec and Cys residues. We began by evaluating the redox status of Trx in RKO colon cancer cells where endogenous TR1 levels were attenuated with siRNA. In these same cells deficient in wild-type TR1, we then induced the expressed a Sec-deficient TR1 and found that this protein altered neither the Trx redox status nor the cellular growth kinetics. Only in cells under oxidative stress from treatment with diamide did we find differences in TR1-comprimised cells. This led us to examine the effects of TR1 knockdown on a variety of oxidative stressors including reactive oxygen species, an electrophilic lipid and a nitric oxide (NO)-prodrug. The effects of TR1 depletion were most pronounced in combination with the latter treatment.

NO has a broad spectrum of physiological effects, including pronounced effects in the vascular and nervous systems [18], [19]. It also is promising as an antineoplastic pharmacological agent due to its cytotoxicity; however, optimal clinical response requires novel delivery mechanisms of the NO to the tumor rather than systemic administration to avoid the vascular adverse effects [20]. *O* ^2^-(2,4-dinitrophenyl) 1-[(4-ethoxycarbonyl) piperazin-1-yl]diazen-1-ium-1,2-diolate (JS-K) is a prodrug designed to release NO intracellularly [21] therefore avoiding generalized effects on the vasculature. The release of NO from JS-K is dependent on metabolism by glutathione S-transferases (GSTs) and this dependency may provide additional neoplastic selectivity since GSTs are frequently overexpressed in cancer [22]. JS-K has demonstrated antineoplastic efficacy in both human cancer cell lines as well as animal model systems [23].

NO can have diverse effects in cells. Functioning as an oxidant, it can react with metal ions, or directly modify proteins on cysteine residues forming S-nitrosothiols. This modification can modulate protein function [24]. The cellular redox management of cysteine nitrosylation is an active area of research, and the TR-Trx system has been identified as a regulator of this phenomenon [25]. In particular, apoptotic proteins have been identified as target proteins modified by nitrosylation [26]. Indeed, the effector caspase, caspase-3, is target of nitrosylation that is modulated by cytosolic and mitochondrial Trx systems [27]. Therefore, NO and the TR-Trx system are integral components in cellular processes of programmed cell death. In the current work we extend the study of this interaction to include the effects of TR1 on the activity of an important new candidate cancer therapeutic agent, JS-K.

## Results

**Figure 1:**
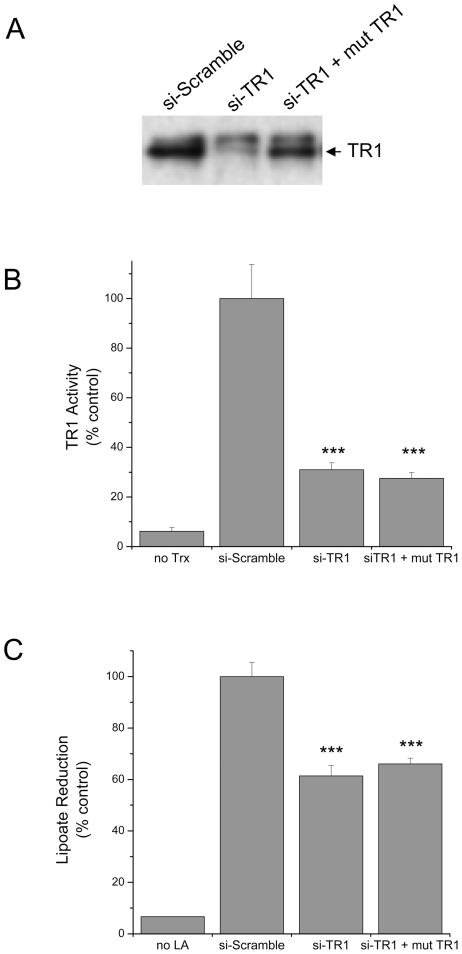
Characterization of TR1 levels and activity in RKO cells. Cells were exposed to siRNA directed against TR1 for a total of 96 hrs, and a Sec-deficient C-terminal mutant TR1 was induced for the last 24 hrs. A) Immunoblot analysis of TR1 protein expression following siRNA treatments and induction of the Sec-deficient mutant TR1. B) TR biochemical activity measured in cell lysates by monitoring NADPH oxidation in an assay that is dependent on Trx and uses insulin is the final electron acceptor. The first bar on the left represent the activity of the control (si-Scramble) without Trx added to the reaction mix, indicating background signal. C) Cell-based TR activity as measured by lipoic acid reduction in a colorimetric assay using Ellman's reagent. The first bar on the left represent the activity of the control (si-Scramble) without lipoic acid added to the reaction mix. The lysates from cells with TR1 knocked down show significant reductions in activity compared to the control (si-Scramble) in both assays (***, p<0.001).

Mammalian thioredoxin reductase without a Sec was thought to have minimal activity; however, recent reports suggest that Sec-deficient thioredoxin reductase might have other redox activities. Therefore, we constructed an inducible cell line where we could express a C-terminal mutant of TR1 that was resistant to siRNA knockdown. We measured the expression of TR1 by Western blotting and measured the TR activity based on insulin reduction as well as lipoic acid reduction (Figure 1). The siRNA effectively knock down the endogenous TR1 by ∼70% and the tetracycline induction of the stably integrated of the C-terminal mutant was ∼75% of the level of the endogenous TR1 (Figure 1A). In addition, the knockdown resulted in ∼70% reduction of TR activity as measured by the biochemical assay of NADPH oxidation with Trx as the intermediate and insulin serving at the final electron acceptor (Figure 1B). Since TR1 can reduce alternative substrates to Trx *in vitro*, we also evaluated the ability of the C-terminal mutant of TR1 to reduce lipoic acid in a cell based assay (Figure 1C). Several cellular enzymes can reduce lipoate but we did observe an ∼40% diminution of lipoate reduction in the RKO cells with endogenous TR1 attenuated by the siRNA and in the cells expressing the C-terminal mutant TR1.

**Figure 2:**
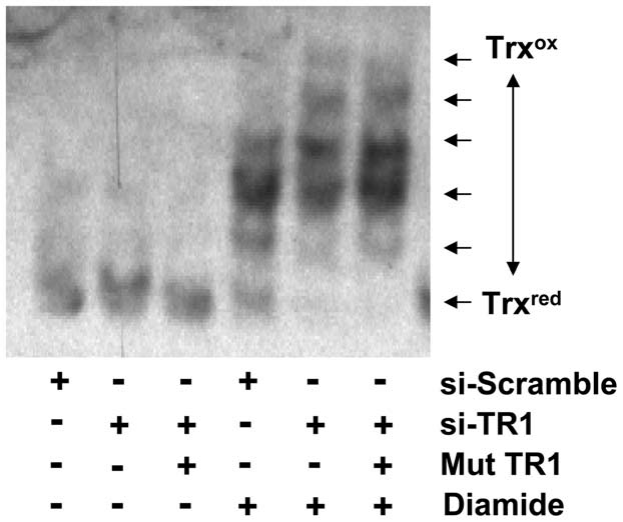
Evaluation of the 6 possible redox states of Trx. In cells without stimulation (left three lanes), Trx is primarily in the reduced state, even with TR1 knocked-down and the C-terminal mutant TR1 expressed. With 1 mM diamide stimulation for 30 min (right three lanes), cells with endogenous TR1 demonstrate more reduced Trx than cells with endogenous TR1 knocked-down with siRNA.

Since Trx is a primary substrate of TR1 and since other Sec-deficient TR1 have demonstrated oxidative stress, we measured the Trx redox status to determine if the Sec-deficient C-terminal mutant TR1 altered the redox status of Trx. Assessment of Trx redox status was performed through alkylation with iodoacetic acid, reduction of oxidized Cys with DTT, and then iodoacetamide alkylation, as has been described [28]. No changes in Trx redox status were observed among the cells with endogenous TR1, cells with TR1 knocked-down, and cells with endogenous TR1 knocked-down plus induction of the Sec-deficient C-terminal mutant TR1 (example dataset in Figure 2 and summary of multiple experiments in Table 1). If the cells were challenged with 1 mM diamide, changes in the redox status of Trx were observed, and a difference between the si-TR1 treated cells and the si-Scramble was evident suggesting that the assay detects alterations in redox status following an oxidative challenge.

**Table 1:** Summary of Trx redox status following TR1 knockdown and Sec-deficient TR1 expression: Percent Trx in each redox state (% total).

Redox state	si-Scramble	si-Scramble+diamide	si-TR1	si-TR1+diamide	si-TR1+mTR1	si-TR1+mTR1+diamide
1 (oxidized)	0.7±0.2	2.9±1.7	1.6±0.4	5±1	3±0.8	3.6±0.6
2	0.7±0.2	11±3.2	1.9±0.6	22±3.8	2.4±0.8	24±4.4
3	1.9±0.4	16±1.3	3.5±1.0	29±4	7±0.7	36±2.8
4	7.6±1.3	42±6.1	8.7±1.1	32±3.3	9±1.9	34±4.4
5	17±1.9	14±2.9	16±2.4	9±0.2	8±0.7	6±1.9
6 (reduced)	75±2.3	21±4.6	68±2.8	4.5±2	76±3.2	4.3±1

Summary of the 6 possible redox states, from the most oxidized (state 1) to the least (state 6) of Trx in RKO cells with endogenous TR1 (si-Scramble), with endogenous TR1 knocked-down (si-TR1), or endogenous TR1 knocked-down but with induced Sec-deficient mutated TR1 (si-TR1+mTR1). Since the majority of the Trx was found to be in the reduced state, we stimulated with 1 mM diamide for 30 min to oxidize the cells and those cells without endogenous TR1 display more oxidized Trx than cells with endogenous TR1.

**Figure 3:**
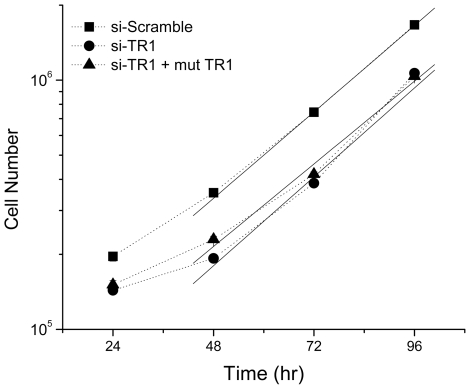
Cell growth kinetics of RKO cells with modulated TR1 levels. A scrambled siRNA was used as a control to measure the basal growth rate (filled square) TR1 was knocked-down by siRNA (filled circle) and with induction of the Sec-deficient, C-terminal mutant TR1 (filled triangle). No significant differences in growth rates were observed as the solid lines used to calculate the growth rates for these conditions are nearly parallel.

Since Sec-deficient TR1 has demonstrated enhanced cytotoxicity in other systems and so one possibility was that the cells with the inducible Sec-deficient TR1 are not proliferating at a similar rate as cells with endogenous TR1. We measured the rate of cell growth following siRNA knockdown and induction of expression the Sec deficient TR1 by counting cell population numbers (Figure 3). The cellular doubling time for all three conditions was ∼24 hrs, following an initial lag period. Therefore, this Sec-deficient TR1 mutant did not appear to alter the growth kinetics of the RKO cells as no significant differences in the slopes of the growth curves were measured (0.35±0.004 cells/hr for si-Scramble, 0.36±0.006 cells/hr for si-TR1, and 0.33±0.008 cells/hr for si-TR1 plus induction of the C-terminal mutant TR1).

**Figure 4:**
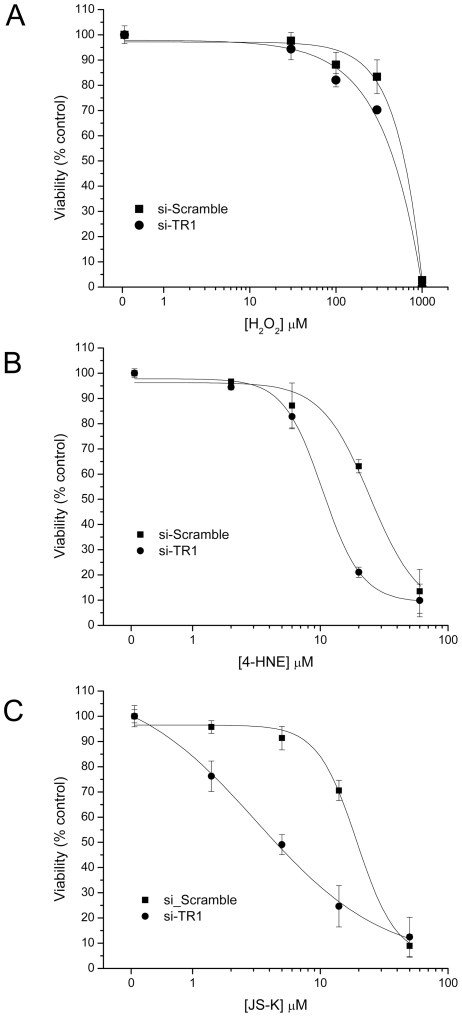
MTT-based viability assays for RKO cells following increasing concentrations of redox modulators. Cells with endogenous TR1 (si-Scramble, filled square), and cells with TR1 knocked-down by siRNA (si-TR1, filled circle) were compared at equivalent doses of the redox modulators. A) RKO cells were treated with increasing concentrations of H_2_O_2_ and the viability was measured after a 24 hrs exposure. No significant differences were observed. B) RKO cells were treated with increasing concentrations of 4-HNE and the viability was measured after a 24 hrs exposure. The si-TR1 cells displayed ∼2-fold increased sensitivity to 4-HNE. C) RKO cells were treated with increasing concentrations of JS-K and the viability was measured after a 24 hrs exposure. The si-TR1 cells displayed ∼6-fold increased sensitivity to JS-K.

Since the induced expression of the Sec-deficient C-terminal mutant TR1 construct did not elicit an alteration in redox status of Trx, we evaluated the cytotoxic response of RKO cells with endogenous TR1 as well as cells where the TR1 was attenuated with siRNA to reactive oxygen and nitrogen in the form of H_2_O_2_, the oxidized lipid 4-HNE, or the NO donor JS-K (Figure 4). Viability following H_2_O_2_ exposure was not different (Figure 4A); the 4-HNE exposure demonstrated a modest, ∼2-fold increased sensitivity in the cells with TR1 knocked-down with a LC_50_ difference of 10.6±0.7 µM in the cells with TR1 knocked down compared to 24±3.4 µM in the cells with endogenous TR1 (Figure 4B); the NO donor, JS-K, demonstrated ∼6-fold increased sensitivity in the cells with TR1 attenuated by siRNA with a LC_50_ difference of 3.1±0.5 µM in the cells with TR1 knocked down compared to 19±2 µM in the cells with endogenous TR1, as measured with a MTT assay (Figure 4C).

**Figure 5:**
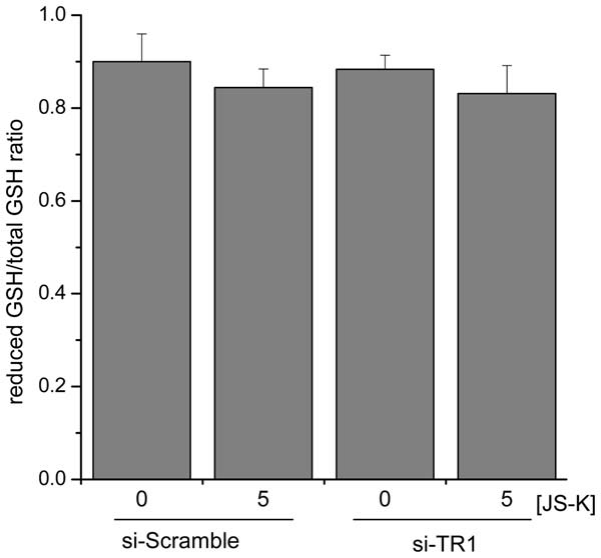
RKO cellular redox state following JS-K treatment as measured by glutathione redox status. GSH measurements were made following treatment with 5 µM JS-K for 24 hrs., and the ratio of the reduced GSH to the total GSH was measured. No significant differences were observed among the treatment groups.

Since the NO-donor promoted a more prominent difference in viability between the RKO cells with endogenous TR1 compared to the cells with TR1 knocked-down, additional evaluation of cellular redox status were performed to evaluate the mechanism of the NO-mediated enhanced cytotoxicity. First, based on the significant differences in cell viability observed in the MTT assay, the Trx redox status was evaluated following 5 µM JS-K incubation. The JS-K treated TR1 knockdown cells displayed a more oxidized distribution of Trx redox states following 90 min incubation with the NO prodrug (Table 2). Next, a more generalized evaluation of the oxidative state of the cells was evaluated following 5 µM JS-K treatment for 24 hrs by measuring the ratio of reduced GSH to the total GSH levels. No significant differences in reduced GSH to total GSH were observed (Figure 5). These data suggest that a global change in redox status was not observed but that select proteins might be targeted.

**Table 2:** Summary of Trx redox status following TR1 knockdown and JS-K treatment: Percent Trx in each redox state (% total).

Redox state	si-Scramble	si-Scramble+JS-K	si-TR1	si-TR1+JS-K
1 (oxidized)	0.3±0.3	0.5±0.5	0.4±0.6	1.4±0.2
2	0.6±0.7	1.9±1.4	0.4±0.4	2.4±2.0
3	1.5±0.3	7.9±2.3	2.1±1.2	15±1.8
4	6.4±1.3	18±3.6	9.9±0.3	32±1.1
5	19±1.8	35±5.6	23±2.8	27±5.8
6 (reduced)	72±3.8	38±5	65±3.5	24±5.4

Summary of the 6 possible redox states of Trx in RKO cells with endogenous TR1 (si-Scramble) or with endogenous TR1 knocked-down (si-TR1) following treatment with 5 µM JS-K for 90 min. Trx does show a shift to more oxidized states in the cells treated with JS-K with TR1 knocked down.

**Figure 6:**
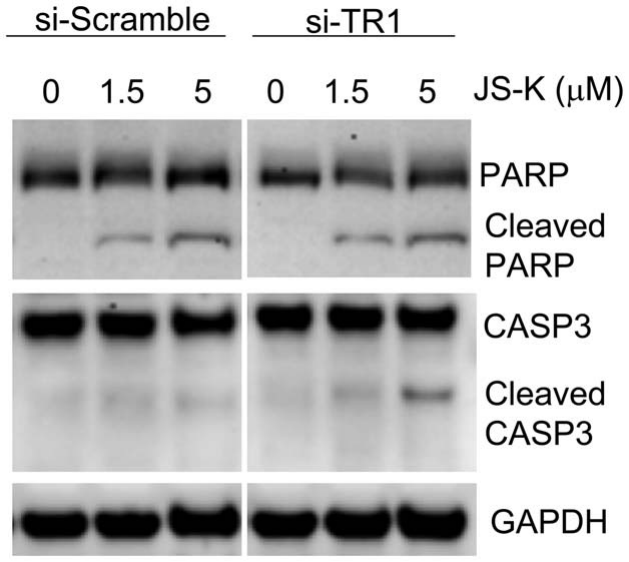
Immunoblot evaluation of cleaved PARP and caspase 3. RKO cells treated with vehicle, 1.5, or 5 µM JS-K for 24 hrs. Protein was separated by SDS-PAGE and detected with immunoblot analysis. A dose-dependent increase in cleaved PARP and caspase 3 (CASP3) was observed with more cleaved material in the TR1 knockdown. GAPDH was evaluated as a loading control.

To determine the mechanism behind the changes in cellular viability as determined by the MTT assay, immunoblot analysis of caspase 3 and the DNA repair protein poly ADP ribose polymerase (PARP) were evaluated (Figure 6). Cleaved caspase 3 is consistent with the initiation of apoptosis and the amount of caspase 3 cleavage appeared to be more extensive when TR1 was knocked-down. Cleaved PARP was also observed in these experiments in a dose dependent manner.

**Figure 7:**
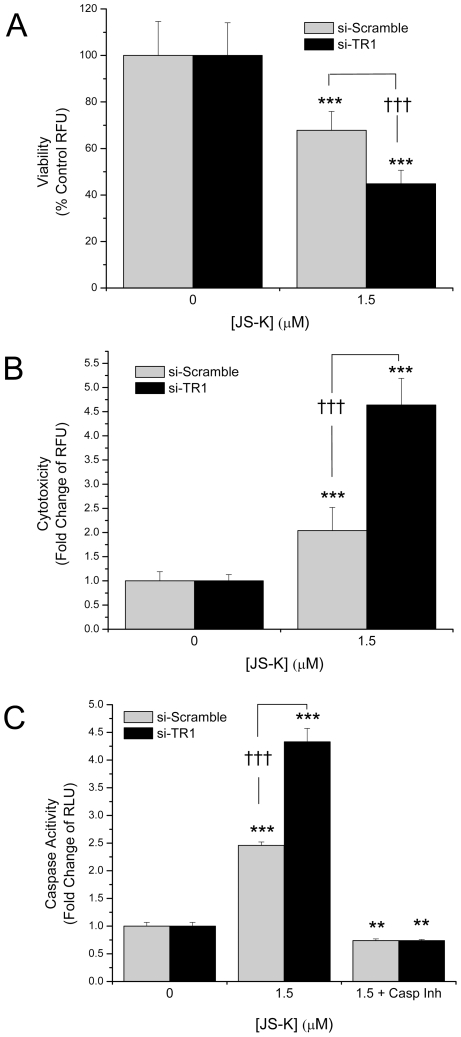
Protease activity as a measure of viability, cytotoxity and effector caspase activity. RKO cells treated with 1.5 µM JS-K for 24 hrs. were assessed for A) viability, B) cytotoxicity, and C) caspase-3/7 activity. In separate experiments, cells were incubated with Z-Asp-CH_2_-DCB, a broad spectrum, competitive caspase inhibitor, to determine that the caspase assay was indeed demonstrating effector caspase activity (C). RKO cells with TR1 knocked-down demonstrate significantly greater losses in viability, increased cytotoxicity, and increased caspase-3/7 activity following JS-K treatment than RKO cells with endogenous TR1 (**, p<0.01; ***, p<0.001; †††, p<0.001).

Evidence of apoptosis initiation was observed at both 1.5 and 5 µM JS-K in the immunoblot analysis; therefore, the cellular viability and cytotoxicity where re-evaluated following 1.5 µM JS-K incubation (where the cells still appear >75% viable, Figure 4) based on protease activity using the MultiTox assay. This assays appeared to be more sensitive than the MTT assay, since even at this low dose, JS-K resulted in significant cytotoxicity and/or loss of viability in the TR1 knockdown cells (∼45% viable) compared to the cells with endogenous levels of TR1 (∼68% viable, Figure 7A and 7B). Next the relative caspase-3/7 activity was measured and consistent with the cytotoxicity data, there was a significant enhancement of caspase activity in the cells with TR1 knocked-down (Figure 7C). In separate experiments, the broad spectrum competitive caspase inhibitor, Z-Asp-CH_2_-DCB, was included during the incubation with JS-K confirming the enzymatic activity previously observed was caspase-dependent activity.

## Discussion

While selenoprotein levels are generally dependent on selenium and selenium deficiency appears to result in increased risk of cancer mortality [29], the TR1-Trx system may be unusual among selenoenzymes in its ability to promote cancer [8], [30]. Indeed, Trx is frequently over expressed in many tumors, may have anti-apoptotic properties, and may contribute to some forms of therapy resistance [7], [31]–[34]. From this perspective, inhibition of TR1 is an excellent target to inhibit the reduction of thioredoxin in the presence of additional oxidative stress. Also, several commonly used therapeutic agents, like cisplatin, cyclophosphamide, and doxorubicin appear to target thioredoxin reductase as well as DNA [35]–[39]. Our results suggest that attenuation of TR1 is insufficient to alter the growth status of cells, but that appropriate redox stress following attenuation of TR1 may be the most effective means of attaining a cytotoxic response.

The antiapoptotic activity of Trx has been sited as rationale for targeting the TR-Trx system in human cancer [40], [41]. The recent observation that the TR-Trx system modulates the activity of caspase-3 in a nitrosylation-dependent manner [27] suggests a prominent role for the TR-Trx system in NO-mediated apoptotic activity. In this work, we also observe that the NO prodrug, JS-K, increases apoptosis when TR1 is knocked down with siRNA (Figures 4 and 6). This mechanism is consistent with previous mechanistic data demonstrating increased caspase activity in acute myelogenous leukemia cells [42]. In addition, NO-donating aspirin demonstrated synergistic activity when combined with gold-containing compounds that are thought to primarily target the TR-Trx system [43].

The role of TR1 in apoptosis has been the subject of investigation since it was identified as a “GRIM” gene (i.e., genes associated with retinoid-IFN-induced mortality, GRIM 12) in a screen for genes related to retinoid-IFN-induced apoptosis [17]. More recently, it has been demonstrated that TR1 protein without a functional Sec residue due to alkylation or truncation, when introduced to cells using *BioPORTER*, induced apoptosis [16], [44]. However, the mechanisms of TR-mediated apoptosis by TR1 SecTRAPs remain unknown. The mutant TR1 we utilized, with the C-terminus Gly-Ser-Ser-Gly, was not a functional thioredoxin reductase as measured by NADPH oxidation/insulin reduction as well as lipoic acid reduction (Figure 1); however, it also did not appear to function as a SecTRAP apoptotic initiator as described by Arnér and colleagues [16]. Similar to a previous report [45], we were unable to identify basal alterations in Trx or cellular redox status when TR1 was knocked-down with siRNA (Figure 2, Table 1) but did observe altered Trx redox status following JS-K treatment (Table 2). In addition, the expression of this Sec-deficient mutant TR1 did not alter the oxidative status of Trx. Therefore, it appears that not all Sec-deficient TR proteins promote oxidative stress and apoptosis.

Targeting TR1 for cancer therapy may not be without undesirable adverse effects if it is not targeted at the tumor. For example, the tumor suppressor, p53, is an important regulator of cell growth and apoptosis, and TR1 enhances p53 function, presumably by contributing reducing equivalents through Trx to the nuclear redox regulator Ref-1 [12], [13], [46], [47]. Several commonly used therapeutic agents, like cisplatin, cyclophosphamide, and doxorubicin appear to target TR1 as well as DNA [35]-[39], but perhaps, a cause for some of the adverse effects observed with these common therapeutics, may be the “off-target” inhibition of TR1. Another redox modulatory compound that has been evaluated in cancer clinical trials, motexafin gadolinium, was thought to specifically target TR1 [7]. However, motexafin gadolinium appears to be a substrate for TR1 and generates reactive oxygen species through this interaction as well as being an inhibitor of ribonucleotide reductase [48]. If this compound's clinical activity is truly due to its interactions with TR1, it is still unclear which tumors should be targeted since this compound has demonstrated mixed results in clinical trials to date [49]–[51], but it appears to hold particular promise as a radiation sensitizer [52], [53].

Even with potential complications of targeting TR1 in cancers, the results herein suggest that drug combination approaches, like the NO-donor, JS-K, might be most effective if combined with agents that target TR1.

## Materials and Methods

### Materials

Advanced DMEM, Glutamax, 5,5′,6,6′-tetrachloro-1,1′,3,3′-tetraethylbenzimidazolyl-carbocyanine iodide (JC-1, MitoProbe JC-1 Assay Kit), 3-(4,5-dimethylthiaxol-2-yl)-2,5-diphenyltretraxolium bromide (MTT), Hank's balanced salt solution with Ca and Mg (HBSS), Tris-glycine 8% gels and bovine serum albumin were purchased from Invitrogen (Carlsbad, CA). Fetal bovine serum was purchased from Hyclone (Logan, UT). The RKO cell line was purchased from American Tissue Type Culture Collection (Manassas, VA). Monoclonal antibodies directed against thioredoxin reductase (B-2, sc-28321, lot# J1304); polyclonal antibodies directed against thioredoxin (FL-105, sc-20146, lot# A1907), and GAPDH (FL-335, sc-25778); donkey polyclonal anti-mouse and anti-rabbit antibodies conjugated with horseradish peroxidase were purchased from Santa Cruz Biotechnology (Santa Cruz, CA). Additional polyclonal antibodies directed against caspase 3 (9661), cleaved caspase 3 (9662), and PARP (9532) were purchased from Cell Signaling Technology (Beverly, MA). Bovine serum albumin standard and Coomassie Plus Protein Reagent were from Pierce Biotechnology (Rockford, IL). Protease inhibitor cocktail tablets (complete®) were purchased from Roche (Indianapolis, IN). PVDF membrane was purchased from Millipore (Burlington, MA). The caspase inhibitor, Z-Asp-CH_2_-DCB, was from Peptides International (Louisville, KY). Western Lighting chemiluminescence reagents were from PerkinElmer Life Sciences (Boston, MA). JS-K was synthesized as previously described [54]. Dimethylsulfoxide (DMSO) and common buffers and salts were purchased from Sigma-Aldrich (St. Louis, MO).

### Cell culture

RKO colon cancer cells were used as a representative colon cell line and were maintained in Advanced DMEM supplemented with 1% Glutamax and 2% fetal bovine serum. Previously, we described the site directed mutagenesis of the C-terminus of TR1 from Gly-Cys-Sec-Gly to Gly-Ser-Ser-Gly plus a silent mutation into the siRNA identity site to make this construct resistant to siRNA directed at the endogenous TR1. We subcloned this mutated TR1 construct into pcDNA5/FRT/TO and then inserted into the RKO cells with stably integrated pcDNA6/TR and pFRT/*lac*Zeo, rendering a tetracycline inducible mutant TR1 cell line. These mutations to TR1 as well as the siRNA used to modulate endogenous TR1 were previously described [13]. Experimentally, the cells were plated at 2–3×10^5^ cells/well in 6 well plate and transfected with siRNA directed at TR1 (si-TR1) or control generated by scrambling the si-TR1 sequence (si-Scramble) for 72 hrs. Then, the cells were treated or stimulated with tetracycline for 24 hrs as indicated.

### Immunoblot analysis

Cells in 6-well plates were placed on ice. Media was aspirated and cells were then washed with 1 ml of cold 1× PBS and the PBS aspirated. Cell lysates were collected as previously described [13]. Protein concentrations were determined using Coomassie Plus Protein Reagent (Pierce). Absorbance at 595 nm was measured using a Perkin-Elmer Victor^3^V plate reader. Ten to 15 µg of protein were separated on either 8% Tris-glycine gels (for TR1) or 10% native urea gels (for Trx), transferred to PVDF membrane, blocked with 10% non-fat dry milk, incubated with primary antibody (1∶200 for TR, 1∶250 for Trx, 1∶1000 for both caspase 3 and cleaved caspase 3, 1∶1000 for PARP, and 1∶500 for GAPDH) overnight at 4°C, washed 3×, incubated with secondary antibody (1∶5000) for 45 min at 22°C, washed 3×, incubated with chemiluminesence reagents, and exposed to x-ray film.

### Thioredoxin reductase activity assays

Cellular TrxR1 activity was measured as has been previously described [13]. In addition, we measured lipoic acid reduction similar to a previously described assay [55]. Briefly, cells were plated and treated as described. Then the cells were trypsinized, washed with PBS, and resuspended in a solution of 1 ml containing 5 mM glucose in PBS, with or without 1 mM lipoic acid and 0.2 mM DTNB with gently shaking at 37°C for 15 min. The cells were centrifuged and the supernatant sample was diluted 1∶1 with water and the absorbance was measured at 412 nm. The negative control was medium with no cells. The cell pellet was washed with PBS, lysed cells in lysis buffer and the protein measured using the Bradford assay. The reduced lipoate was normalized by the cellular protein content.

### Redox status of Trx

The redox status of Trx was performed as described [28]. Briefly, cells were lysed in 8 M urea buffered with Tris to pH 8.9 containing 30 mM iodoacetic acid, sonicated, and incubated for 15 min at 37°C. Protein was precipated with 10 volumes of ice cold acetone-1N HCl (98∶2, vol/vol), centrifuged at 11,000×g for 5 min at 4°C, washed with cold acetone-HCl, resuspended in 95 µl of buffered urea containing 35 mM DTT, incubated for 30 min at 37°C, 7.5 µl of 200 mM iodoacetamide was add to each sample, and incubated for 15 min at 37°C. Protein concentration was estimated using Coomassie Plus Protein Reagent.

### MTT assay

Cellular viability was determined using an MTT as previously described [56], which relies on tetrazolium salt reduction by NADH in viable cells (Berridge *et al.*, 2005).

### Glutathione quantitation

Reduced glutathione (GSH) and total GSH were measured using GSH-GLO reagents (Promega). Approximately 2.5×10^3^ cells that were pre-incubated with siRNA directed at TR1 were plated in white sided 384 well plates, allowed to adhere, and then treated with 0 or 5 µM JS-K for 24 hrs. Media was removed by centrifugation, the reduced GSH was directly measured according to manufacturing instructions, and the total GSH was measured by incubating the cells with 1 mM TCEP to reduce oxidized GSH. This assay is a glutathione-S transferase-dependent assay that uses GSH to generate luciferin as a substrate for luciferase to generate light. Luminescence was measured using a Perkin-Elmer Victor^3^V plate reader.

### MultiTox assay

Viability and cytotoxicity measurements were assessed by differential protease activities using the MultiTox-Fluor Multiplex Assay (Promega). This assay uses a GF-AFC substrate that is cell permeable to assess the viable cells, and a bis-AAF-R110 substrate that is not cell permeable to assess protease activity from dead cells. The fluorescence from these substrates were measured using a Perkin-Elmer Victor^3^V plate reader; the GF-AFC viability substrate was measured at 405 nm excitation, 475 nm emission; and the bis-AAF-R110 cytotoxicity substrate was measured at 485 nm excitation, 535 nm emission.

### Caspase activity assay

Effector caspase activity was measured using the Caspase-GLO 3/7 Assay (Promega) according to the manufacturer's instructions. This is an assay where a DEVD peptide substrate is cleaved by active caspases to release aminoluciferin as a substrate for luciferase to produce light. Luminescence was measured using a Perkin-Elmer Victor^3^V plate reader.

### Statistical analysis

1-way ANOVA was used to determine statistical significance among samples (GraphPad InStat Version 3.06). Bonferroni multiple comparisons post hoc testing was used to establish significance among the treatment groups with p<0.05 considered significant.

## References

[1] Rundlof AK, Arner ES (2004). Regulation of the mammalian selenoprotein thioredoxin reductase 1 in relation to cellular phenotype, growth, and signaling events.. Antioxid Redox Signal.

[2] Spyrou G, Holmgren A (1996). Deoxyribonucleoside triphosphate pools and growth of glutathione-depleted 3T6 mouse fibroblasts.. Biochem Biophys Res Commun.

[3] Spyrou G, Bjornstedt M, Kumar S, Holmgren A (1995). AP-1 DNA-binding activity is inhibited by selenite and selenodiglutathione.. FEBS Lett.

[4] Hu J, Ma X, Lindner DJ, Karra S, Hofmann ER (2001). Modulation of p53 dependent gene expression and cell death through thioredoxin-thioredoxin reductase by the Interferon-Retinoid combination.. Oncogene.

[5] Huang LE, Arany Z, Livingston DM, Bunn HF (1996). Activation of hypoxia-inducible transcription factor depends primarily upon redox-sensitive stabilization of its alpha subunit.. J Biol Chem.

[6] Matthews JR, Wakasugi N, Virelizier JL, Yodoi J, Hay RT (1992). Thioredoxin regulates the DNA binding activity of NF-kappa B by reduction of a disulphide bond involving cysteine 62.. Nucleic Acids Res.

[7] Biaglow JE, Miller RA (2005). The thioredoxin reductase/thioredoxin system: novel redox targets for cancer therapy.. Cancer Biol Ther.

[8] Yoo MH, Xu XM, Carlson BA, Patterson AD, Gladyshev VN (2007). Targeting thioredoxin reductase 1 reduction in cancer cells inhibits self-sufficient growth and DNA replication.. PLoS ONE.

[9] Bjornstedt M, Hamberg M, Kumar S, Xue J, Holmgren A (1995). Human thioredoxin reductase directly reduces lipid hydroperoxides by NADPH and selenocystine strongly stimulates the reaction via catalytically generated selenols.. J Biol Chem.

[10] Arner ES, Nordberg J, Holmgren A (1996). Efficient reduction of lipoamide and lipoic acid by mammalian thioredoxin reductase.. Biochem Biophys Res Commun.

[11] Nordberg J, Arner ES (2001). Reactive oxygen species, antioxidants, and the mammalian thioredoxin system.. Free Radic Biol Med.

[12] Moos PJ, Edes K, Cassidy P, Massuda E, Fitzpatrick FA (2003). Electrophilic prostaglandins and lipid aldehydes repress redox-sensitive transcription factors p53 and hypoxia-inducible factor by impairing the selenoprotein thioredoxin reductase.. J Biol Chem.

[13] Cassidy PB, Edes K, Nelson CC, Parsawar K, Fitzpatrick FA (2006). Thioredoxin reductase is required for the inactivation of tumor suppressor p53 and for apoptosis induced by endogenous electrophiles.. Carcinogenesis.

[14] Fang J, Holmgren A (2006). Inhibition of thioredoxin and thioredoxin reductase by 4-hydroxy-2-nonenal in vitro and in vivo.. J Am Chem Soc.

[15] Lothrop AP, Ruggles EL, Hondal RJ (2009). No selenium required: reactions catalyzed by mammalian thioredoxin reductase that are independent of a selenocysteine residue.. Biochemistry.

[16] Anestal K, Prast-Nielsen S, Cenas N, Arner ES (2008). Cell death by SecTRAPs: thioredoxin reductase as a prooxidant killer of cells.. PLoS One.

[17] Hofmann ER, Boyanapalli M, Lindner DJ, Weihua X, Hassel BA (1998). Thioredoxin reductase mediates cell death effects of the combination of beta interferon and retinoic acid.. Mol Cell Biol.

[18] Lamas S, Perez-Sala D, Moncada S (1998). Nitric oxide: from discovery to the clinic.. Trends Pharmacol Sci.

[19] Moncada S, Palmer RM, Higgs EA (1991). Nitric oxide: physiology, pathophysiology, and pharmacology.. Pharmacol Rev.

[20] Moncada S, Higgs EA (2006). The discovery of nitric oxide and its role in vascular biology.. Br J Pharmacol.

[21] Shami PJ, Saavedra JE, Wang LY, Bonifant CL, Diwan BA (2003). JS-K, a glutathione/glutathione S-transferase-activated nitric oxide donor of the diazeniumdiolate class with potent antineoplastic activity.. Mol Cancer Ther.

[22] Kiziltepe T, Hideshima T, Ishitsuka K, Ocio EM, Raje N (2007). JS-K, a GST-activated nitric oxide generator, induces DNA double-strand breaks, activates DNA damage response pathways, and induces apoptosis in vitro and in vivo in human multiple myeloma cells.. Blood.

[23] Shami PJ, Saavedra JE, Bonifant CL, Chu J, Udupi V (2006). Antitumor activity of JS-K [O2-(2,4-dinitrophenyl) 1-[(4-ethoxycarbonyl)piperazin-1-yl]diazen-1-ium-1,2-diolate] and related O2-aryl diazeniumdiolates in vitro and in vivo.. J Med Chem.

[24] Hess DT, Matsumoto A, Kim SO, Marshall HE, Stamler JS (2005). Protein S-nitrosylation: purview and parameters.. Nat Rev Mol Cell Biol.

[25] Stoyanovsky DA, Tyurina YY, Tyurin VA, Anand D, Mandavia DN (2005). Thioredoxin and lipoic acid catalyze the denitrosation of low molecular weight and protein S-nitrosothiols.. J Am Chem Soc.

[26] Kim KM, Kim PK, Kwon YG, Bai SK, Nam WD (2002). Regulation of apoptosis by nitrosative stress.. J Biochem Mol Biol.

[27] Benhar M, Forrester MT, Hess DT, Stamler JS (2008). Regulated protein denitrosylation by cytosolic and mitochondrial thioredoxins.. Science.

[28] Bersani NA, Merwin JR, Lopez NI, Pearson GD, Merrill GF (2002). Protein electrophoretic mobility shift assay to monitor redox state of thioredoxin in cells.. Methods Enzymol.

[29] Bleys J, Navas-Acien A, Guallar E (2008). Serum selenium levels and all-cause, cancer, and cardiovascular mortality among US adults.. Arch Intern Med.

[30] Yoo MH, Xu XM, Carlson BA, Gladyshev VN, Hatfield DL (2006). Thioredoxin reductase 1 deficiency reverses tumor phenotype and tumorigenicity of lung carcinoma cells.. J Biol Chem.

[31] Kakolyris S, Giatromanolaki A, Koukourakis M, Powis G, Souglakos J (2001). Thioredoxin expression is associated with lymph node status and prognosis in early operable non-small cell lung cancer.. Clin Cancer Res.

[32] Shao L, Diccianni MB, Tanaka T, Gribi R, Yu AL (2001). Thioredoxin expression in primary T-cell acute lymphoblastic leukemia and its therapeutic implication.. Cancer Res.

[33] Grogan TM, Fenoglio-Prieser C, Zeheb R, Bellamy W, Frutiger Y (2000). Thioredoxin, a putative oncogene product, is overexpressed in gastric carcinoma and associated with increased proliferation and increased cell survival.. Hum Pathol.

[34] Nilsson J, Soderberg O, Nilsson K, Rosen A (2000). Thioredoxin prolongs survival of B-type chronic lymphocytic leukemia cells.. Blood.

[35] Bracht K, Liebeke M, Ritter CA, Grunert R, Bednarski PJ (2007). Correlations between the activities of 19 standard anticancer agents, antioxidative enzyme activities and the expression of ATP-binding cassette transporters: comparison with the National Cancer Institute data.. Anticancer Drugs.

[36] Witte AB, Anestal K, Jerremalm E, Ehrsson H, Arner ES (2005). Inhibition of thioredoxin reductase but not of glutathione reductase by the major classes of alkylating and platinum-containing anticancer compounds.. Free Radic Biol Med.

[37] Ravi D, Das KC (2004). Redox-cycling of anthracyclines by thioredoxin system: increased superoxide generation and DNA damage.. Cancer Chemother Pharmacol.

[38] Wang X, Zhang J, Xu T (2007). Cyclophosphamide as a potent inhibitor of tumor thioredoxin reductase in vivo.. Toxicol Appl Pharmacol.

[39] Arivarasu NA, Fatima S, Mahmood R (2007). Effect of cisplatin on brush border membrane enzymes and anti-oxidant system of rat intestine.. Life Sci.

[40] Lincoln DT, Ali Emadi EM, Tonissen KF, Clarke FM (2003). The thioredoxin-thioredoxin reductase system: over-expression in human cancer.. Anticancer Res.

[41] Arner ES, Holmgren A (2006). The thioredoxin system in cancer.. Semin Cancer Biol.

[42] Udupi V, Yu M, Malaviya S, Saavedra JE, Shami PJ (2006). JS-K, a nitric oxide prodrug, induces cytochrome c release and caspase activation in HL-60 myeloid leukemia cells.. Leuk Res.

[43] Sun Y, Rigas B (2008). The thioredoxin system mediates redox-induced cell death in human colon cancer cells: implications for the mechanism of action of anticancer agents.. Cancer Res.

[44] Anestal K, Arner ES (2003). Rapid induction of cell death by selenium-compromised thioredoxin reductase 1 but not by the fully active enzyme containing selenocysteine.. J Biol Chem.

[45] Watson WH, Heilman JM, Hughes LL, Spielberger JC (2008). Thioredoxin reductase-1 knock down does not result in thioredoxin-1 oxidation.. Biochem Biophys Res Commun.

[46] Moos PJ, Edes K, Fitzpatrick FA (2000). Inactivation of wild-type p53 tumor suppressor by electrophilic prostaglandins.. Proc Natl Acad Sci U S A.

[47] Seemann S, Hainaut P (2005). Roles of thioredoxin reductase 1 and APE/Ref-1 in the control of basal p53 stability and activity.. Oncogene.

[48] Hashemy SI, Ungerstedt JS, Zahedi Avval F, Holmgren A (2006). Motexafin gadolinium, a tumor-selective drug targeting thioredoxin reductase and ribonucleotide reductase.. J Biol Chem.

[49] Amato RJ, Jac J, Hernandez-McClain J (2008). Motexafin Gadolinium for the Treatment of Metastatic Renal Cell Carcinoma: Phase II Study Results.. Clin Genitourin Cancer.

[50] William WN, Zinner RG, Karp DD, Oh YW, Glisson BS (2007). Phase I trial of motexafin gadolinium in combination with docetaxel and cisplatin for the treatment of non-small cell lung cancer.. J Thorac Oncol.

[51] Ford JM, Seiferheld W, Alger JR, Wu G, Endicott TJ (2007). Results of the phase I dose-escalating study of motexafin gadolinium with standard radiotherapy in patients with glioblastoma multiforme.. Int J Radiat Oncol Biol Phys.

[52] Richards GM, Mehta MP (2007). Motexafin gadolinium in the treatment of brain metastases.. Expert Opin Pharmacother.

[53] Rosenberg A, Knox S (2006). Radiation sensitization with redox modulators: a promising approach.. Int J Radiat Oncol Biol Phys.

[54] Saavedra JE, Srinivasan A, Bonifant CL, Chu J, Shanklin AP (2001). The secondary amine/nitric oxide complex ion R(2)N[N(O)NO](-) as nucleophile and leaving group in S9N)Ar reactions.. J Org Chem.

[55] May JM, Qu ZC, Nelson DJ (2007). Uptake and reduction of alpha-lipoic acid by human erythrocytes.. Clin Biochem.

[56] Mullally JE, Moos PJ, Edes K, Fitzpatrick FA (2001). Cyclopentenone prostaglandins of the J series inhibit the ubiquitin isopeptidase activity of the proteasome pathway.. J Biol Chem.

